# Data-Aided SNR Estimation for Bandlimited Optical Intensity Channels

**DOI:** 10.3390/s22228660

**Published:** 2022-11-09

**Authors:** Wilfried Gappmair

**Affiliations:** Institute of Communication Networks and Satellite Communications, Graz University of Technology, Inffeldgasse 12, 8010 Graz, Austria; gappmair@tugraz.at

**Keywords:** SNR estimation, optical wireless communications, intensity modulation

## Abstract

Not only for radio frequency but also for optical communication systems, knowledge of the signal-to-noise ratio (SNR) is essential, e.g., for an adaptive network, where modulation schemes and/or error correction methods should be selected according to the varying channel states. In the current paper, this topic is discussed for a bandlimited optical intensity link under the assumption that the data symbols are known to the receiver unit in form of pilot sequences. This requires a unipolar signal design regarding the symbol constellation, but also a non-negative pulse shape satisfying the Nyquist criterion is necessary. Focusing on this kind of scenario, the modified Cramer–Rao lower bound is derived, representing the theoretical limit of the error performance of the data-aided SNR estimator developed in this context. Furthermore, we derive and analyze a maximum likelihood algorithm for SNR estimation, which turns out to be particularly simple for specific values of the excess bandwidth, among them the most attractive case of minimum bandwidth occupation. Numerical results confirming the analytical work conclude the paper.

## 1. Introduction

In a series of papers recently published by the author [1,2,3], parameter estimation and synchronization for a bandlimited optical intensity link have been discussed. In this context, a unipolar waveform design is indispensable with respect to pulse shaping and symbol constellation [4,5]. Furthermore, it is most helpful that pulse shapes satisfy the Nyquist criterion, which allows for a simple detection process in the receiver unit [6].

However, not only for radio frequency (RF) but also for optical wireless communication (OWC) solutions [7,8,9,10], the relevant transmission parameters have to be recovered reliably, because otherwise subsequent receiver stages, such as error correction algorithms, cannot be operated in an efficient way [11,12]. In particular, recovery of the symbol timing is of paramount importance in this respect, since this is a prerequisite for many other estimation and detection procedures. In [1,2,3], it has been shown how this might be achieved for a bandlimited optical intensity link under different conditions, e.g., whether data are known to the receiver unit or not in the form of pilot sequences, or if the estimator or synchronizer module is to be implemented in a feedforward or feedback manner.

Usually, the estimation of the signal-to-noise ratio (SNR) requires that the symbol timing has been established before by a properly selected algorithm. It is to be recalled that knowledge of the SNR is normally needed for adaptive communication systems to select modulation and coding schemes according to the given channel conditions [13], but also powerful error correction methods—such as turbo or LDPC algorithms—need this sort of information [14]. Scanning the open literature, numerous papers are available about SNR estimation in RF channels, e.g., the frequently cited overview by Pauluzzi and Beaulieu [15], but little or no information is published for OWC systems. This has been the main motivation of the current contribution addressing data-aided SNR estimation for a bandlimited optical intensity channel. Finally, it is to be noticed that the article was prepared for a Special Issue of Feature Papers 2022 in the Communications Section of MDPI Sensors.

The rest of the paper is organized as follows: the signal and channel model for analytical and simulation work is introduced in Section 2, whereas Section 3 focuses on the derivation of the Cramer–Rao lower bound (CRLB) as the theoretical limit of the jitter performance for any algorithm discussed in the context of SNR estimation. In Section 4, we derive a maximum likelihood (ML) algorithm and analyze it in terms of mean value and variance. Numerical results are shown in Section 5, and Section 6 concludes the paper.

## 2. Signal and Channel Model

As already mentioned in the introductory section, properly selected pulse shapes and modulation schemes are necessary to satisfy the non-negativity as well as the Nyquist constraints required for a bandlimited optical intensity channel. In this respect, it is assumed that the real-valued data symbols *a_k_*, k∈ℤ, are independent and identically distributed (i.i.d.) elements of an *M*-ary PAM alphabet A. It makes sense to organize the alphabet such that the symbols are normalized to unit energy, i.e., $E[a_{k}^{2}]=1$, where E[⋅] denotes the expectation operator. Then, with $\eta_{M}=\frac{1}{6}(M-1)(2M-1)$, we have $a_{k}\in A$ = $\frac{1}{\sqrt{\eta_{M}}}\{0,\;1,\;\ldots ,\;M-1\}$. This means that the average value is given by

(1) $$\mu_{a}=E[a_{k}]=\frac{1}{\sqrt{\eta_{M}}}\;\frac{M-1}{2}=\sqrt{\frac{3\;(M-1)}{2\;(2M-1)}}$$

On the other hand, the signal at the output of the opto-electrical receiver module is obtained as

(2) $$r(t)=A\;\sum_{k}a_{k\;}h(t-kT-\tau )+w(t)$$

where *A* > 0 is the channel gain, *h*(*t*) describes the pulse shape, *T* and *τ* symbolize the symbol period and the propagation delay between receiver and transmitter station, respectively. We assume that *A* is a constant regarding the observation interval used for estimation purposes, because variations of the channel state are normally slow enough so that fading effects need not be taken into account. As already required previously, *h*(*t*) must satisfy the non-negativity as well as the Nyquist criterion, e.g., achieved by a squared raised cosine function [1,6]. In line with the investigations carried out in [1,2,3,4,5,6], the receiver signal in (2) is also assumed to be distorted by additive white Gaussian noise (AWGN), in the following expressed by *w*(*t*), with zero mean and variance $\sigma_{w}^{2}$.

In addition, we introduce the average optical power as $P_{0}=\mu_{a\;}\bar{h}$, where

(3) $$\bar{h}=\frac{1}{\sqrt{T}}\int_{-\infty }^{\infty }h(t)\;dt$$

so that the average electrical SNR at the receiver can be defined as

(4) $$\gamma_{s}=\frac{A^{2}P_{0}^{2}}{\sigma_{w}^{2}}$$

**Figure 1:**
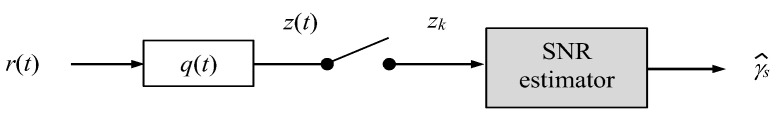
Signal model for SNR estimation.

However, before being treated in further stages of operation, the signal in (2) has to pass the receiver filter *q*(*t*), whose output is given by z(t)= q(t)⊗r(t), where ⊗ denotes the convolutional operator. For convenient reasons, this is summarized in Figure 1.

Since there exists no simple solution for a matched filter structure in the context of a bandlimited optical intensity link [6], it is suggested that *q*(*t*) exhibits a flat behavior over the spectrum occupied by the user component in (2). This straightforward approach guarantees that the waveform will not be distorted, but the price to be paid is an increased amount of noise the subsequent receiver stages have to cope with. In particular, this means that the transfer function of the filter performs a rectangular shape in the frequency domain, i.e., $Q(f)=F[q(t)]=\sqrt{T}$ for |f| ≤(1+α)/T and Q(f)=0 elsewhere, with *α* as the roll-off factor (excess bandwidth) of the selected pulse shape; recall that *α* = 0 indicates the minimum bandwidth scenario. The related impulse response is then furnished by application of the inverse Fourier transform [16], i.e.,

(5) $$q(t)=F^{-1}[Q(f)]=\frac{2(1+\alpha )}{\sqrt{T}}\mathrm{sinc}[2(1+\alpha )t/T]$$

with sinc(*x*) = sin(πx)/(πx). Of course, the noise signal at this flat filter output develops as *n*(*t*) = *w*(*t*) ⊗ *q*(*t*) representing a zero-mean non-white Gaussian process. Assuming in the next step that the symbol timing has been reliably recovered and corrected, e.g., by the algorithm proposed in [1], the *T*-spaced samples at the output of the receiver filter are obtained as

(6) $$z_{k}=z(kT)=A\cdot a_{k}+n_{k\;}$$

where $E[n_{k}]=0$ and $E[n_{i\;}n_{k}]=2(1+\alpha )\;\sigma_{w}^{2}\;\mathrm{sinc}[2(1+\alpha )(i-k)]$.

## 3. Cramer–Rao Lower Bound

### 3.1. Derivation of the Log-Likelihood Function

The Cramer–Rao lower bound (CRLB) is a major figure of merit when it comes to the estimation of a parameter [17]. The reason behind this is the fact that the bound represents the theoretical limit of the jitter (error) variance of any estimator developed in this context.

According to the signal model specified previously, we have to consider the parameter vector **u** = (*u*_1_, *u*_2_) = (*A*, *σ_w_*). The CRLB for *u_i_* is determined by

(7) $$\mathrm{CRLB}(u_{i})={\left[J^{-1}(u)\right]}_{i}$$

where [·]*_i_* indicates the *i*-th diagonal entry of the inverted Fisher information matrix (FIM) expressed by **J**(**u**). In the case that no nuisance parameter needs to be taken into account, the FIM entries are computed as

(8) $$J_{i,k}\equiv {\left[J(u)\right]}_{i,k}=-E\left[\frac{\partial^{2}\Lambda (z;u)}{\partial u_{i\;}\partial u_{k}}\right]$$

with **z** as the given vector of observables, Λ(**z**;**u**) denotes the log-likelihood function (LLF) characterizing the communication link, and E[⋅] symbolizes expectation with respect to the noise model.

By inspection of (8), it is clear that the computation of the CRLB requires the knowledge of the LLF describing the subject of investigation. To this end, we assume that a sequence of *L* receiver samples (6) forms the vector **z** expressed by

(9) z=A⋅a+n

The vector **a** of known data symbols specifies the pilot sequence, which is to be used for estimation purposes in the sequel, and **n** denotes the noise vector with covariance matrix

(10) $$R=E[n\cdot n^{T}]=2(1+\alpha )\;\sigma_{w}^{2}\;\Omega$$

where the entries for line *i* and column *k* of **Ω** are given by $\omega_{ik}=\mathrm{sinc}[2(1+\alpha )(i-k)]=\omega_{ki}$ forming this way a symmetric Toeplitz matrix [18]. As a result, the likelihood function for our estimation problem can be written as [19,20],

(11) $$\mathrm{Pr}(z;u)=\frac{1}{\sqrt{{\left(2\pi \right)}^{L}\det(R)}}e^{-\frac{1}{2}\;{\left(z-A\;a\right)}^{T}R^{-1}(z-A\;a)}$$

However, instead of using **u** = (*A*, *σ_w_*), it is easier to concentrate in the following on the average electrical SNR normalized by $P_{0}^{2}$, i.e., $\rho_{s}=\gamma_{s}/P_{0}^{2}=A^{2}/\sigma_{w}^{2}$. Then, by introduction of $P_{n}=\sigma_{w}^{2}$, we have that **u** = (*ρ_s_*, *P_n_*) and the related LLF is furnished by

(12) $$\Lambda (z;u)=\log\mathrm{Pr}(z;u)\sim -\frac{L}{2}\log P_{n}-\frac{z^{T}\Psi \;z-2\sqrt{\rho_{s}P_{n}}\;z^{T}\Psi \;a+\rho_{s}P_{n}\;a^{T}\Psi \;a}{4(1+\alpha )P_{n}}$$

which has been achieved by $\Psi =\Omega^{-1}$ as well as omitting all immaterial constants and factors not depending on **u**.

### 3.2. Modified Cramer–Rao Lower Bound

In the next step, the FIM entries are obtained by computing the second-order derivatives according to (8), the results of which have then to be averaged with respect to the noise vector **n**. However, this approach means that the CRLB will be a function of the selected pilot sequence **a**. Therefore, it is suggested to extend the averaging procedure to **a** as well, which creates the so-called modified Cramer–Rao lower bound (MCRLB) [21,22,23]. Doing so, we get after some algebra:

(13) $$J_{11}=-E\left[\frac{\partial^{2}\Lambda (z;u)}{\partial \rho_{s}^{2}}\right]=\frac{1}{8(1+\alpha )\rho_{s}}E_{a}[a^{T}\Psi \;a]$$

(14) $$J_{22}=-E\left[\frac{\partial^{2}\Lambda (z;u)}{\partial P_{n}^{2}}\right]=-\frac{L}{2P_{n}^{2}}+\frac{\rho_{s}}{8(1+\alpha )P_{n}^{2}}E_{a}[a^{T}\Psi \;a]+\frac{1}{2(1+\alpha )P_{n}^{3}}E_{n}[n^{T}\Psi \;n]$$

(15) $$J_{12}=J_{21}=-E\left[\frac{\partial^{2}\Lambda (z;u)}{\partial \rho_{s}\partial P_{n}}\right]=\frac{1}{8(1+\alpha )P_{n}}E_{a}[a^{T}\Psi \;a]$$

Evaluating (7) for *u*_1_ = *ρ_s_*, the corresponding MCRLB is given by

(16) $$\mathrm{MCRLB}(\rho_{s})=\frac{J_{22}}{J_{11}J_{22}-J_{12}^{2}}$$

Substituting (13)–(15) into (16) and scaling the result with respect to $\rho_{s}^{2}$, we obtain the normalized MCRLB expressed as

(17) $$\begin{array}{cc} \mathrm{NMCRLB}(\rho_{s}) & =\frac{\mathrm{MCRLB}(\rho_{s})}{\rho_{s}^{2}}\\ & =2(1+\alpha )\left(\frac{P_{n}}{E_{n}[n^{T}\Psi \;n]-L(1+\alpha )P_{n}}+\frac{4}{\rho_{s}\;E_{a}[a^{T}\Psi \;a]}\right) \end{array}$$

Introducing the auxiliary terms

(18) $${\bar{\Psi }}_{0}=\frac{1}{L}\sum_{i=0}^{L-1}\psi_{ii},\;{\bar{\Psi }}_{1}=\frac{1}{L}\sum_{i=0}^{L-1}\sum_{k=i+1}^{L-1}\psi_{ik},\;{\bar{\Psi }}_{2}=\frac{1}{L}\sum_{i=0}^{L-1}\sum_{k=0}^{L-1}\omega_{ik}\;\psi_{ik}$$

where *ψ_ik_* is the entry of **Ψ** indicating line *i* and column *k*, the expected operations in (17) can be written as

(19) $$\begin{array}{cc} E_{a}[a^{T}\Psi \;a] & =\sum_{i=0}^{L-1}\sum_{k=0}^{L-1}E[a_{i\;}a_{k}]\;\psi_{ik}\\ & =\sum_{i=0}^{L-1}E[a_{i}^{2}]\;\psi_{ii}+\sum_{i=0}^{L-1}\sum_{k=0,k\neq i}^{L-1}E[a_{i}a_{k}]\;\psi_{ik}\\ & =\eta_{a}\sum_{i=0}^{L-1}\psi_{ii}+2\mu_{a}^{2}\sum_{i=0}^{L-1}\sum_{k=i+1}^{L-1}\psi_{ik}=L({\bar{\Psi }}_{0}+2\mu_{a}^{2}{\bar{\Psi }}_{1}) \end{array}$$

and

(20) $$\begin{array}{cc} E_{n}[n^{T}\Psi \;n] & =\sum_{i=0}^{L-1}\sum_{k=0}^{L-1}E[n_{i}n_{k}]\;\psi_{ik}\\ & =\sigma_{n}^{2}\sum_{i=0}^{L-1}\sum_{k=0}^{L-1}\omega_{ik}\;\psi_{ik}=2L(1+\alpha )P_{n\;}{\bar{\Psi }}_{2} \end{array}$$

Finally, by plugging (19) and (20) into (17), we have that

(21) $$\mathrm{NMCRLB}(\rho_{s})=\frac{2}{L}\left(\frac{1}{2{\bar{\Psi }}_{2}-1}+\frac{4(1+\alpha )}{\rho_{s}({\bar{\Psi }}_{0}+2\mu_{a}^{2}{\bar{\Psi }}_{1})}\right)$$

Nevertheless, the relationship might be simplified for $\alpha \in \{0,\;\frac{1}{2},\;1\}$, because in this case $\omega_{ik}=\;\mathrm{sinc}[2(1+\alpha )(i-k)]=1$ for *i* = *k* and zero elsewhere. This means that $\Omega =\Omega^{-1}$ = $\Psi =I_{L}$, with **I***_L_* as the *L*-dimensional identity matrix, which means also that ${\bar{\Psi }}_{0}={\bar{\Psi }}_{2}=1$ and ${\bar{\Psi }}_{1}=0$. Hence, the normalized bound boils down to

(22) $$\mathrm{NMCRLB}(\rho_{s})=\frac{2}{L}\left(1+\frac{4\;(1+\alpha )}{\rho_{s}}\right)$$

## 4. Maximum Likelihood Estimation

### 4.1. Derivation of the Estimator Algorithm

By means of the LLF in (12), we are basically in the position to derive a maximum likelihood (ML) algorithm for SNR estimation. However, the SNR parameter is composed of two ingredients—channel gain *A* and the noise power *P_n_*—the estimates of which are needed to compute the SNR estimate. This is simply achieved by substituting $\rho_{s}=A^{2}/P_{n}$ into (12), deriving the resulting LLF with respect to *A* and *P_n_*, equating both relationships to zero and solving them analytically. Doing this, we obtain for **u** = (*A*, *P_n_*)

(23) $${\left.\frac{\partial \Lambda (z;u)}{\partial A}\right|}_{u=\hat{u}}=\frac{z^{T}\Psi \;a-\hat{A}\;a^{T}\Psi \;a}{2(1+\alpha ){\hat{P}}_{n}}=0$$

and

(24) $${\left.\frac{\partial \Lambda (z;u)}{\partial P_{n}}\right|}_{u=\hat{u}}=-\frac{L}{2{\hat{P}}_{n}}+\frac{z^{T}\Psi \;z-2\hat{A}\;z^{T}\Psi \;a+{\hat{A}}^{2}a^{T}\Psi \;a}{4(1+\alpha ){\hat{P}}_{n}^{2}}=0$$

Then, by introduction of $M_{aa}=a^{T}\Psi \;a$, $M_{az}=z^{T}\Psi \;a$, and $M_{zz}=z^{T}\Psi \;z$, we find the estimates for channel gain and noise power in closed form:

(25) $$\hat{A}=\frac{z^{T}\Psi \;a}{a^{T}\Psi \;a}=\frac{M_{az}}{M_{aa}}$$

(26) $${\hat{P}}_{n}=\frac{z^{T}\Psi \;z-2\hat{A}\;z^{T}\Psi \;a+{\hat{A}}^{2}a^{T}\Psi \;a}{2(1+\alpha )L}=\frac{1}{2(1+\alpha )L}\left(M_{zz}-\frac{M_{az}^{2}}{M_{aa}}\right)$$

By inspection of (25) and (26), it is clear that a viable solution is only achievable for *M_aa_* > 0, i.e., a pilot sequence consisting of zero symbols only would not work. Finally, according to the invariance principle for ML estimates [24], the SNR solution is given by

(27) $${\hat{\rho }}_{s}=\frac{{\hat{A}}^{2}}{{\hat{P}}_{n}}$$

### 4.2. Probability Analysis

In this subsection, we want to derive the probability density function (PDF) of the SNR estimate in (27) and analyze it in terms of bias and variance. It turns out that this is possible in closed form for **Ψ** = **I***_L_*, i.e., $\alpha \in \{0,\;\frac{1}{2},\;1\}$, whereas for other values of *α*, it is verified in Section 5 that the analytical results achieved with **Ψ** = **I***_L_* are very close to the true ones obtained by numerical means.

By plugging **Ψ** = **I***_L_* into (25), the estimate for the channel gain develops as

(28) $$\hat{A}=\frac{z^{T}a}{a^{T}a}=\frac{{\left(A\;a+n\right)}^{T}a}{a^{T}a}=A+\frac{n^{T}a}{a^{T}a}=A+\frac{1}{M_{aa}}\sum_{k=0}^{L-1}a_{k}n_{k}$$

which means that $\hat{A}$ is a zero-mean Gaussian variate. Computing the variance of the latter, we have to consider that the noise samples *n_k_* are zero-mean and i.i.d. in case that **Ψ** = **I***_L_*. Therefore,

(29) $$\sigma_{A}^{2}=E[{\left(\hat{A}-A\right)}^{2}]=\frac{1}{M_{aa}^{2}}E\left[{\left(\sum_{k=0}^{L-1}a_{k}n_{k}\right)}^{2}\right]=\frac{1}{M_{aa}^{2}}E\left[\sum_{k=0}^{L-1}a_{k}^{2}n_{k}^{2}\right]=\frac{\sigma_{n}^{2}}{M_{aa}}$$

where $\sigma_{n}^{2}=E[n_{k}^{2}]=2(1+\alpha )\;\sigma_{w}^{2}$. The related PDF is then straightforwardly given by

(30) $$f_{A}(\hat{A})=\frac{1}{\sqrt{2\pi }\sigma_{A}}e^{-{\left(\hat{A}-A\right)}^{2}/2\sigma_{A}^{2}}$$

On the other hand, $Y={\hat{A}}^{2}$ corresponds to a non-central Gamma variate [19] characterized by the distribution

(31) $$f_{Y}(y)=\frac{1}{\sqrt{2\pi y\;}\sigma_{A}}e^{-\left(y+A^{2}\right)/2\sigma_{A}^{2}}\cosh\left(\frac{A\sqrt{y}}{\sigma_{A}^{2}}\right)\;,\;y>0$$

If we consider in the next step the estimate of the noise power for **Ψ** = **I***_L_*, we have

(32) $${\hat{P}}_{n}=\frac{z^{T}z-2\hat{A}\;z^{T}a+{\hat{A}}^{2}a^{T}a}{2(1+\alpha )L}$$

With z=A⋅a+n and $\hat{A}$ determined by (28), the numerator in (32) simplifies to

(33) $${\left(A\;a+n\right)}^{T}(A\;a+n)-2\hat{A}\;{\left(A\;a+n\right)}^{T}a+{\hat{A}}^{2}a^{T}a=n^{T}n-\frac{{\left(n^{T}a\right)}^{2}}{a^{T}a}$$

which represents a central Gamma variate with variance $\sigma_{n}^{2}$ and *L*—1 degrees of freedom [20,25]. Hence, by introduction of $X={\hat{P}}_{n}$, the PDF of (32) can be written as

(34) $$f_{X}(x)=\frac{1}{{\left(2\sigma_{x}^{2}\right)}^{\frac{L-1}{2}}\Gamma (\frac{L-1}{2})}x^{\frac{L-1}{2}-1}e^{-x/2\sigma_{x}^{2}},\;x\geq 0$$

where $\sigma_{x}^{2}=\frac{\sigma_{n}^{2}}{2(1+\alpha )L}=\frac{\sigma_{w}^{2}}{L}$. Employing in the following the PDFs in (31) and (34), the distribution of the SNR estimate is determined by (A2) derived in the Appendix A, i.e.,

(35) f(ρ^s)=K0ρ^s(ρ^s+β)L e−λF11L2, 12; λ ρ^sρ^s+β , ρ^s>0

Regarding (A3) and (A4), the parameters *β*, *λ*, and *K*_0_ are functions of the true SNR value denoted by *ρ_s_*, the roll-off factor *α*, the observation length *L*, as well as the selected pilot sequence **a**.

By means of the relationships (A9) and (A10) detailed in the Appendix A, we can specify the first- and second-order moments of (35) as follows:

(36) $$E[{\hat{\rho }}_{s}]=\int_{0}^{\infty }{\hat{\rho }}_{s}\;f({\hat{\rho }}_{s})\;d{\hat{\rho }}_{s}=\frac{L}{L-3}\left(\rho_{s}+\frac{2(1+\alpha )}{M_{aa}}\right)$$

(37) $$E[{\hat{\rho }}_{s}^{2}]=\int_{0}^{\infty }{\hat{\rho }}_{s}^{2}\;f({\hat{\rho }}_{s})\;d\rho_{s}=\frac{L^{2}}{\left(L-3)(L-5\right)}\left(\rho_{s}^{2}+\frac{12(1+\alpha )\rho_{s}}{M_{aa}}+\frac{12{\left(1+\alpha \right)}^{2}}{M_{aa}^{2}}\right)$$

Therefore, bias and variance of ${\hat{\rho }}_{s}$, normalized by $\rho_{s}$ and $\rho_{s}^{2}$, respectively, are given by

(38) $$\mathrm{NBias}({\hat{\rho }}_{s})=\frac{E[{\hat{\rho }}_{s}]-\rho_{s}}{\rho_{s}}=\frac{L}{L-3}\left(1+\frac{2(1+\alpha )}{M_{aa}\rho_{s}}\right)-1$$

and

(39) $$\begin{array}{cc} \mathrm{NVar}({\hat{\rho }}_{s}) & =\frac{E[{\hat{\rho }}_{s}^{2}]-E^{2}[{\hat{\rho }}_{s}]}{\rho_{s}^{2}}\\ & =\frac{2L^{2}}{{\left(L-3\right)}^{2}(L-5)}\left(1+\frac{4(1+\alpha )(L-2)}{M_{aa}\rho_{s}}+\frac{4{\left(1+\alpha \right)}^{2}(L-2)}{M_{aa}^{2}\rho_{s}^{2}}\right) \end{array}$$

Via *M_aa_*, it is obvious that (38) and (39) depend on the selected pilot sequence. In order to avoid this drawback, we could average the relationships with respect to **a**. The problem in this context is that there exists no closed form solution. A way out of this dilemma is Jensen’s inequality [26] (Appendix 1B), which provides us with

(40) $$E[\frac{1}{M_{aa}}]\geq \frac{1}{E[M_{aa}]}=\frac{1}{LE[a_{k}^{2}]}=\frac{1}{L}$$

and

(41) $$E[\frac{1}{M_{aa}^{2}}]\geq \frac{1}{E[M_{aa}^{2}]}=\frac{1}{LE[a_{k}^{4}]+L(L-1)E^{2}[a_{k}^{2}]}=\frac{1}{L\kappa_{a}+L(L-1)}$$

where *κ_a_* denotes the symbol kurtosis of the PAM alphabet, which is given by

(42) $$\kappa_{a}=E[a_{k}^{4}]=\frac{6}{5}\cdot \frac{3M(M-1)-1}{\left(2M-1)(M-1\right)}$$

By taking into account the auxiliary results in (40) and (41), we finally obtain

(43) $$\bar{\mathrm{NBias}}({\hat{\rho }}_{s})=\frac{L}{L-3}\left(1+\frac{2(1+\alpha )}{L\rho_{s}}\right)-1$$

and

(44) $$\bar{\mathrm{NVar}}({\hat{\rho }}_{s})=\frac{2L^{2}}{{\left(L-3\right)}^{2}(L-5)}\left(1+\frac{4(1+\alpha )(L-2)}{L\rho_{s}}+\frac{4{\left(1+\alpha \right)}^{2}(L-2)}{[L\;\kappa_{a}+L(L-1)]\;\rho_{s}^{2}}\right)$$

as lower bounds for the relationships in (38) and (39), respectively.

## 5. Numerical Results

The analytical results achieved for SNR estimation in Section 3 and Section 4 will be verified by Monte Carlo (MC) simulations. In the following, the former are indicated by lines, whereas the latter are shown by markers. Each point in the diagrams below has been obtained by averaging a number of 10^5^ estimates, which turned out to be large enough to verify the analytical results with sufficient accuracy.

**Figure 2:**
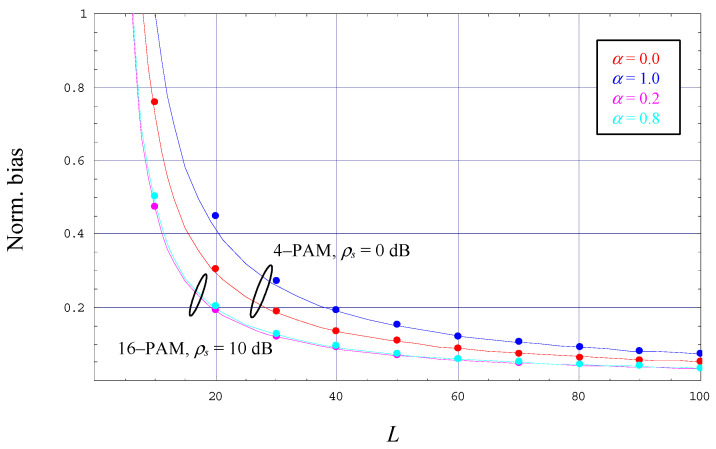
Evolution of the normalized bias.

Assuming a 4-PAM constellation operated with *ρ_s_* = 0 dB and a roll-off factor *α* ∈ {0.0, 1.0}, Figure 2 illustrates the evolution of the normalized bias as a function of the observation length *L*. It is to be noticed that the lines in different colors represent the lower bound given by (43); verified by simulation results, we observe that the lower limit is very tight over the full range of *L*. We observe that the bias decreases rapidly with increasing values of *L*, which is also confirmed by (43). In addition, the diagram depicts the results obtained for 16-PAM, *ρ_s_* = 10 dB, and *α* ∈ {0.2, 0.8}. In the strict sense, the relationship in (43) applies only to values of *α* ∈ {0.0, 0.5, 1.0}, but the 16-PAM scenario in Figure 2 demonstrates that it represents also a very good approximation for other values of the excess bandwidth.

The evolution of the normalized bias has been simulated and verified for modulation schemes other than 4-PAM and 16-PAM, e.g., 2-PAM and 8-PAM, as well as for roll-off factors different to those exemplified in Figure 2. It turned out that the bias of the estimator algorithm is reflected accurately enough by the formula in (43), disappearing for very large values of *L* irrespective of the selected values of *M* or *α*.

**Figure 3:**
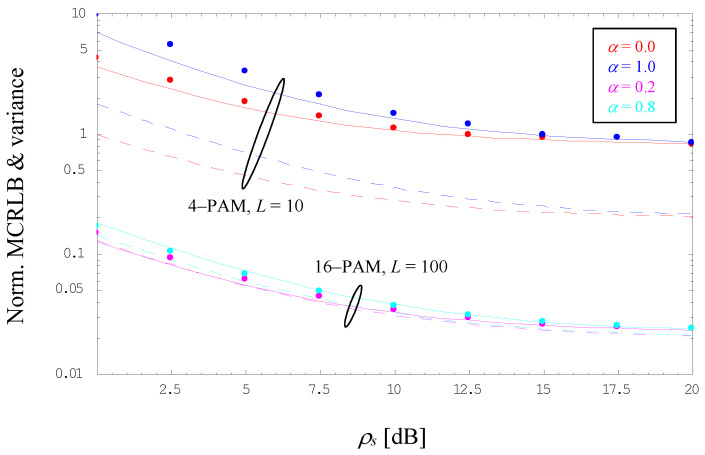
Evolution of the normalized MCRLB and variance.

Using a 4-PAM scheme with *L* = 10 and the same roll-off factors as before, Figure 3 illustrates the evolution of the normalized variance as a function of the true SNR value in dB. For comparison purposes, the normalized MCRLB expressed by (22) is shown in dashed style. We observe that the latter is fairly loose for such small observation windows, whereas the lower bound of the variance in (44) appears to be very tight as confirmed by simulation results, in particular at larger SNR values. However, the diagram illustrates also that the MCRLB is more and more approximated by the jitter variance of the related estimator algorithm, when we increase the observation length in Figure 3, verified for 16-PAM, *L* = 100, and *α* ∈ {0.2, 0.8}; it is to be recalled that for *α* ∉ {0.0, 0.5, 1.0}, the NMCRLB is furnished by (21). Finally, we see that the MC output is very close to (44) over the full SNR range, although the relationship is, in a strict sense, only applicable to roll-off factors *α* ∈ {0.0, 0.5, 1.0}. These observations also hold true for modulation schemes and roll-off factors other than those used in Figure 3; especially, one can see that for *L* ≫ 1 and *ρ_s_* ≫ 1, the normalized variance is simply given by 2/*L*.

## 6. Concluding Remarks

Assuming a data-aided situation, i.e., data symbols are known to the receiver in the form of a pilot sequence, SNR estimation for a bandlimited optical intensity link has been investigated in the current paper. This requires a signal design achieved by an *M*-ary PAM scheme and a non-negative pulse shape also satisfying the Nyquist criterion. By means of a flat receiver filter, it is avoided that the waveforms of the user signal are distorted, but the price to be paid is an additional amount of noise which the subsequent receiver stages are suffering from.

Conditioned on reliable recovery and correction of the symbol timing, the modified CRLB could be derived as the theoretical limit of the jitter variance produced by the SNR estimator developed in the context of this paper. With respect to the latter, an ML solution has been obtained in closed form, which turned out to be particularly simple from a computational point of view for specific values of excess bandwidth, among them being the minimum bandwidth scenario. For these values, the analytical relationships for bias and jitter variance have been obtained in closed form as well.

Verified by simulation results, it could be shown that—irrespective of the chosen PAM constellation and the value of the excess bandwidth—the bias effect vanishes more and more with increasing values of the true SNR value and the observation length *L* the link is operated with. This is also confirmed in view of jitter performance insofar as the CRLB is successively approached by increasing values of *L*.

## Data Availability

Data are available from the author upon mail request.

## Appendix A

Using the identities introduced in Section 4, i.e., $X={\hat{P}}_{n}$ as well as $Y={\hat{A}}^{2}$, together with $Z={\hat{\rho }}_{s}=Y/X$, the PDF of the SNR estimate can be expressed as [27]

(A1) $$f_{Z}(z)=\int_{0}^{\infty }f_{Z|X}(z|x)f_{X}(x)\;dx=\int_{0}^{\infty }x\;f_{Y}(x\;z)f_{X}(x)\;dx$$

Substituting in the sequel the PDFs given by (31) and (34), we obtain by means of [28] (3.462/1) and (9.240) after some lengthy but straightforward manipulations,

(A2) fZ(z)=K0z(z+β)L e−λF11L2, 12; λzz+β

where F11(⋅) denotes the confluent hypergeometric (Kummer) function [29] and the parameters *β*, *λ*, and *K*_0_ are specified as follows:

(A3) $$\lambda =\frac{A^{2}}{2\sigma_{A}^{2}}=\frac{M_{aa}\rho_{s}}{4(1+\alpha )},\;\beta =\frac{\sigma_{A}^{2}}{\sigma_{x}^{2}}=\frac{2(1+\alpha )L}{M_{aa}}$$

(A4) $$K_{0}=\frac{\Gamma (\frac{L}{2})}{\sqrt{\pi }\;\Gamma (\frac{L-1}{2})}\;{\left(\frac{\sigma_{A}^{2}}{\sigma_{x}^{2}}\right)}^{\left(L-1\right)/2}=\frac{\Gamma (\frac{L}{2})}{\sqrt{\pi }\;\Gamma (\frac{L-1}{2})}\beta^{\left(L-1\right)/2}$$

Deriving the *m*-th order moment of (A2), we first express the confluent hypergeometric function by its Meijer G-equivalent [30] (8.4.45/2), i.e.,

(A5) F11(a,b; z)=Γ(b)Γ(a)G2,11,1−1z  1, ba

Applying then the integration rules for Meijer G-functions [30] (2.24.2/6), we get

(A6) $$\begin{array}{cc} M_{m} & =\int_{0}^{\infty }z^{m}f_{Z}(z)\;dz\;,\;m\in {\mathbb{N}}_{0}\\ & =\frac{K_{0}\;\Gamma (\frac{1}{2})}{\Gamma (\frac{L}{2})}\;e^{-\lambda }\int_{0}^{\infty }\frac{z^{m-1/2}}{{\left(z+\beta \right)}^{L/2}}G_{2,1}^{1,1}\left(-\left.\frac{z+\beta }{\lambda z}\;\right|\;\begin{array}{c} \;1,\;\frac{1}{2}\\ \frac{L}{2} \end{array}\right)\;dz\\ & =\;K_{0}\frac{\;\Gamma (\frac{1}{2})\;\Gamma (\frac{L-2m-1}{2})}{\Gamma (\frac{L}{2})}\beta^{\left(2m+1-L\right)/2}e^{-\lambda }G_{3,2}^{2,1}\left(-\left.\frac{1}{\lambda }\;\right|\;\begin{array}{c} \;1,\;\frac{1}{2},\;\frac{L}{2}\\ m+\frac{1}{2},\;\frac{L}{2} \end{array}\right) \end{array}$$

Employing the integral definition for Meijer G-functions [30] (8.2.1/1) as well as the identity in (A5), which means that

(A7) G3,22,1−1λ   1, 12, L2m+12, L2=G2,11,1−1λ   1, 12m+12=Γ(m+12)Γ(12)F11m+12, 12; λ

the relationship in (A6) might be simplified to

(A8) Mm=K0Γ(m+12) Γ(L−2m−12)Γ(L2)β(2m+1−L)/2e−λF11m+12, 12; λ

Finally, by taking into account the properties of confluent hypergeometric functions, the first- and second-order moments are provided as

(A9) $$M_{1}=\frac{\sqrt{\pi }K_{0}}{2}\frac{\;\Gamma (\frac{L-3}{2})}{\Gamma (\frac{L}{2})}\beta^{-(L-3)/2}(2\lambda +1)$$

and

(A10) $$M_{2}=\frac{\sqrt{\pi }K_{0}}{4}\frac{\;\Gamma (\frac{L-5}{2})}{\Gamma (\frac{L}{2})}\beta^{-(L-5)/2}(4\lambda^{2}+12\lambda +3)$$
